# Left atrial strain predicts paroxysmal atrial fibrillation recurrence after catheter ablation: a 1-year study using three-dimensional speckle-tracking echocardiography

**DOI:** 10.1186/s12872-024-04447-0

**Published:** 2025-02-04

**Authors:** Rui Zhang, He Li, Yan Wang, Tianle Yu, Jiacheng Li, Yumeng Wu, Zhiwen Yu, Cuixing Liang, Dan Yu, Li Xue

**Affiliations:** Department of Cardiovascular Ultrasound, the Fourth Affiliated Hospital of Harbin Medical University, Harbin, China

**Keywords:** Three-dimensional speckle tracking echocardiography (3DSTE), Paroxysmal atrial fibrillation, Radiofrequency catheter ablation (RFCA), Strain

## Abstract

**Background:**

Radiofrequency catheter ablation (RFCA) is a widely employed method for restoring sinus rhythm(SR) in patients with drug-refractory paroxysmal atrial fibrillation (PAF). Three-dimensional speckle tracking echocardiography (3DSTE) is a precise and practical imaging technique for clinically assessing myocardial function in the left atrium. The objective of this study was to assess alterations in three-dimensional strains and predict recurrence in patients with PAF following RFCA.

**Methods:**

A total of 109 patients diagnosed with drug-refractory PAF and scheduled for RFCA were included in this study between September 2019 and June 2022. Conventional echocardiography and 3DSTE were performed prior to and one year(median period of 12.2 months) after RFCA. Global three-dimensional left atrial (LA) strain parameters, along with those of the left ventricle, were measured and analyzed statistically. The primary study endpoint was the recurrence of atrial fibrillation (AF).

**Results:**

Among the 109 patients, 78 maintained a stable SR during the one-year follow-up after RFCA, while 31 experienced a recurrence of AF. Notably, patients who sustained SR demonstrated significant improvements in various LA strain parameters, including reservoir, pump, and conduit functions, compared to both their preoperative levels and those of patients who experienced recurrence(*p* < 0.05). Additionally, patients with sustained SR exhibited a significant reduction in LA volume compared to those with recurrence(*p* = 0.003). Furthermore, left ventricular global longitudinal strain (LVGLS) and left ventricular global area strain (LVGAS) of the left ventricle showed improvement while maintaining a preserved left ventricular ejection fraction (LVEF) after RFCA(*p* < 0.05). Our multivariate regression analysis revealed that left atrial reservoir strain (LASr) independently predicted the recurrence of AF [odds ratio (OR), 1.19, 95% confidence interval (CI), 1.05–1.35, *p* = 0.005]. Receiver operating characteristic(ROC) curve showed that the area under the curve(AUC) for LASr in assessing the risk of recurrence after RFCA in patients with PAF was 0.70 ( 95% CI, 0.60–0.81, *P* = 0.001). The calculated cutoff value was 16.5%.

**Conclusions:**

RFCA plays a pivotal role in preserving SR and restoring LA function in patients with PAF. 3D-STE is highly effective for post-RFCA prognostic assessment. LASr, a predictive marker for the recurrence of PAF assists in the stratification of risk and contributes to informed treatment decisions, offering valuable points of reference.

## Introduction

Atrial fibrillation (AF) is the most prevalent form of supraventricular arrhythmia, frequently progressing from its initial paroxysmal state to a persistent form. Elevated ventricular rates in AF are associated with symptoms like palpitations, fatigue, and decreased physical endurance, which can hasten disease progression and potentially lead to irreversible heart failure (HF) [1]. Therefore, managing ventricular rate is crucial in treating AF [2]. Studies have consistently shown that radiofrequency catheter ablation (RFCA) is a reliable approach for maintaining sinus rhythm (SR), particularly effective in paroxysmal AF (PAF) patients with preserved ejection fraction [3–6].

The left atrium (LA) is integral to cardiac function, facilitating ventricular filling through its reservoir, conduit, and booster pump functions. Dysfunction in these capacities can diminish left ventricular (LV) systolic output. While RFCA is generally effective, recurrence remains a significant concern, with LA size serving as a predictor of AF recurrence post-ablation [7]. Thus, monitoring changes in myocardial mechanics following RFCA is critical for tracking disease progression.

Conventional echocardiography, despite being widely used, lacks sensitivity in detecting subclinical myocardial changes [8]. Two-dimensional speckle tracking echocardiography (2DSTE) measures myocardial deformation; however, it is prone to artifacts related to cardiac cycle variations. In contrast, three-dimensional speckle tracking echocardiography (3DSTE) captures comprehensive three-dimensional data in a single cardiac cycle, thereby overcoming the limitations of 2DSTE. This technology exhibits high sensitivity in detecting early myocardial changes, even in patients with preserved left ventricular ejection fraction (LVEF).

Currently, 3DSTE primarily focuses on evaluating LA structure and function using volume and strain metrics. Despite its potential, research on its application to LA evaluation remains limited [9]. This study aims to determine the effectiveness of 3DSTE in assessing LA structure and function after RFCA and to explore its prognostic value in predicting postoperative recurrences. The findings aim to improve clinical management and risk stratification for PAF patients.

## Materials and methods

### Study population

**Fig. 1:**
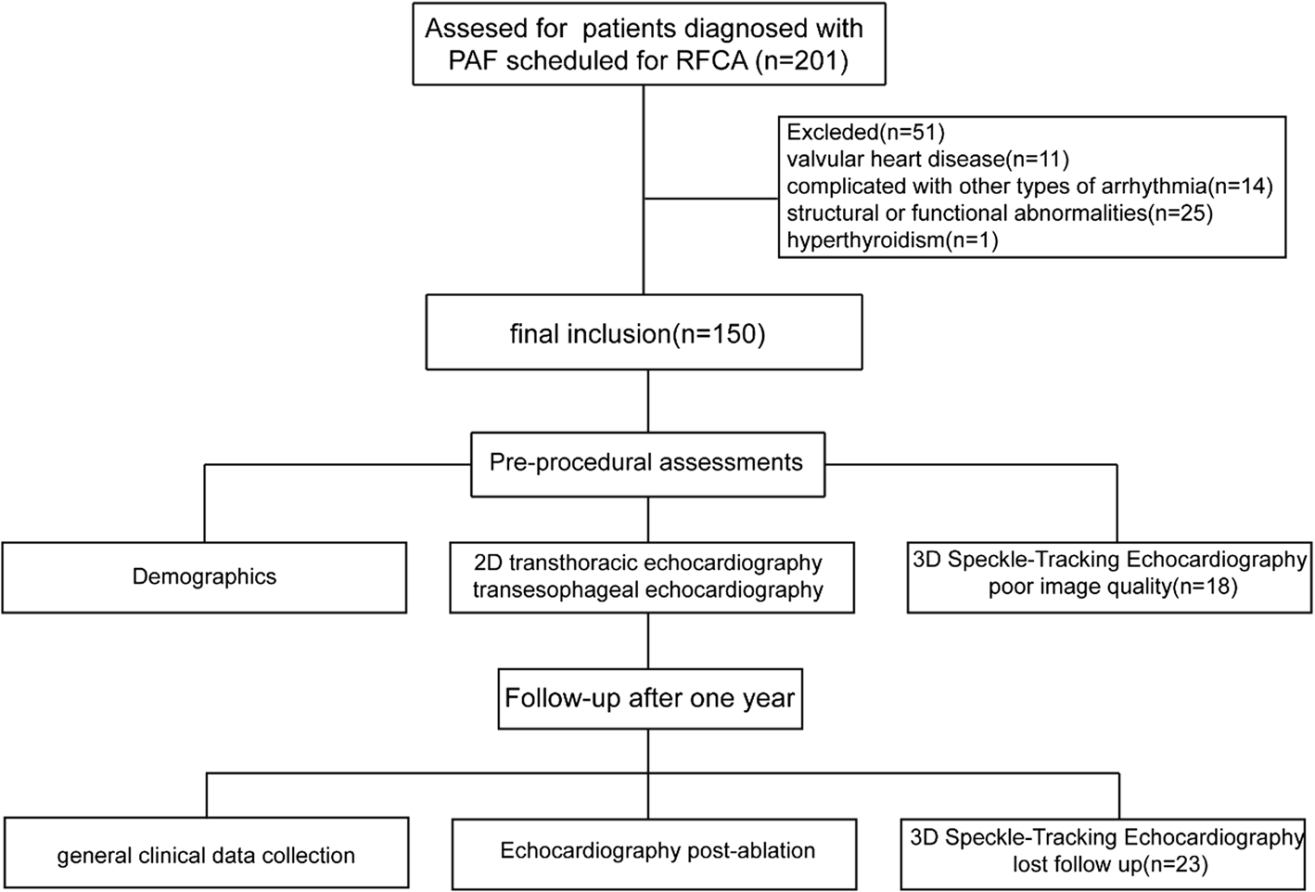
Study flow

This single-center prospective study enrolled 201 consecutive patients with drug-refractory PAF scheduled for their first RFCA at the Fourth Affiliated Hospital of Harbin Medical University between June 2019 and July 2022. All patients were in SR during echocardiographic acquisition. The inclusion criteria, following the ESC atrial fibrillation management guidelines, were as follows: 1. Patients with AF episodes lasting less than 7 days, not requiring anti-arrhythmic medication or electrical cardioversion for conversion to SR.2. P-wave disappearance and replacement by F-waves, with a frequency of 350–600 beats/min and irregular RR intervals. 3. LVEF ≥ 50% calculated by modified Simpson’s biplane rule [10, 11]. Exclusion criteria were: Patients with valvular heart disease, complicated with other types of arrhythmia, cardiac structural or functional abnormalities, hyperthyroidism, poor image quality, and lost of follow-up. Enrolled participants were followed up via telephone after one year to verify postoperative recurrence or te maintenance of SR, as well as the feasibility of conducting echocardiography. Two-dimensional (2D) transthoracic echocardiography (TTE) and 3DSTE were performed before and follow-up time after one year after RFCA. Patients who developed arrhythmias after the three-month blanking period underwent routine electrocardiograms and Holter tests based on their symptoms, with the presence of atrial tachycardia for more than 30 s being considered a recurrence. In addition to this, telephone and outpatient follow-ups were performed every three months until their last available time. The follow-up assessment includes evaluating changes in the patient’s daily activities and heart-related symptoms, monitoring medication adherence, and analyzing the occurrence and characteristics of new AF episodes. Regular ECG or Holter monitoring is recommended every 3, 6, and 9 months to detect silent AF and other arrhythmias. If AF recurrence is detected, patients are advised to return for further diagnostic tests. Silent AF, which may not show symptoms, requires continuous monitoring for accurate detection. Follow-up patients were in SR during the image acquisition, while patients experiencing a recurrence of persistent AF required electrical cardioversion two weeks before the ultrasound. After applying the exclusion criteria, a total of 109 patients were ultimately included in the study (Fig. 1). The general clinical data were recorded preoperatively. All AF patients underwent transesophageal echocardiography to exclude the LA appendage and thrombus. The approval of the study protocol was acquired by the Ethics Committee of Harbin Medical University(2023-YXLLSC-07). Moreover, the present study was according to the ethical guidelines of the 1975 Declaration of Helsinki.

Patients were under general anesthesia for the procedure. A steerable catheter was inserted via the femoral vein into the coronary sinus. A transseptal puncture is performed to access the LA from the right atrium. An 8.5 F sheath is advanced into the LA, and heparin is administered to maintain the activated clotting time (ACT) between 250 and 300 s to prevent thrombus formation and air embolism. Pulmonary vein angiography is conducted to visualize the pulmonary veins. A 3D electroanatomical map of the LA is created using mapping systems (Carto3; Biosense Webster) combination with high-density multipolar mapping catheter (PentaRay™, Biosense Webster), which aids in identifying and guiding ablation of areas with abnormal electrical activity. Circumferential pulmonary vein isolation (CPVI) is performed using an irrigated, contact force-sensing ablation catheter(Smarttouch SF, Biosense Webster). The catheter delivers radiofrequency energy, typically with a target temperature of 43 °C and power settings between 30 and 35 watts, to create lesions that block electrical conduction from the pulmonary veins. If AF continues, specific LA areas with abnormal electrical signals (low-amplitude or fractionated electrograms) also be ablated. In cases where AF recurs due to non-pulmonary vein (non-PV) triggers, additional substrate ablation in the LA may be required. To confirm successful isolation, 20 mg adenosine triphosphate (ATP) is administered to test for reconnections between the pulmonary veins and the LA. If linear lesions are created, a bidirectional conduction block is confirmed through pacing maneuvers. The endpoint is reached when AF terminates and normal heart rhythm is restored.

The TTE study was carried out based on a Vivid E95 ultrasound system with an M5Sc-D probe (1.5–4.6 MHz) and 4V-D (1.7–3.3 MHz) probe to collect conventional parameters and 3D echocardiography. All images and 3-lead electrocardiograms were recorded during SR. Furthermore, left atrial diameter (LAD), left ventricular end-diastolic diameter (LVEDd), and left ventricular end-systolic diameter (LVEDs) were measured from the LV long-axis view. Peak early diastole mitral flow velocity (E) was recorded by pulsed-wave Doppler echocardiography in an apical four-chamber view, and mitral annular septal diastolic velocity (e ‘) was acquired by tissue doppler; E/e’ was calculated. Left atrial volume, left ventricular end-diastolic volume (LVEDV), left ventricular end-systolic volume (LVESV), and left ventricular ejection fraction (LVEF) were measured by the modified Simpson’s biplane rule. Additionally, the left atrial volume index (LAVI) was calculated by left atrial volume divided by body surface area. Left ventricular stroke volume (LVSV) was defined as LVEDV- LVESV.

**Fig. 2:**
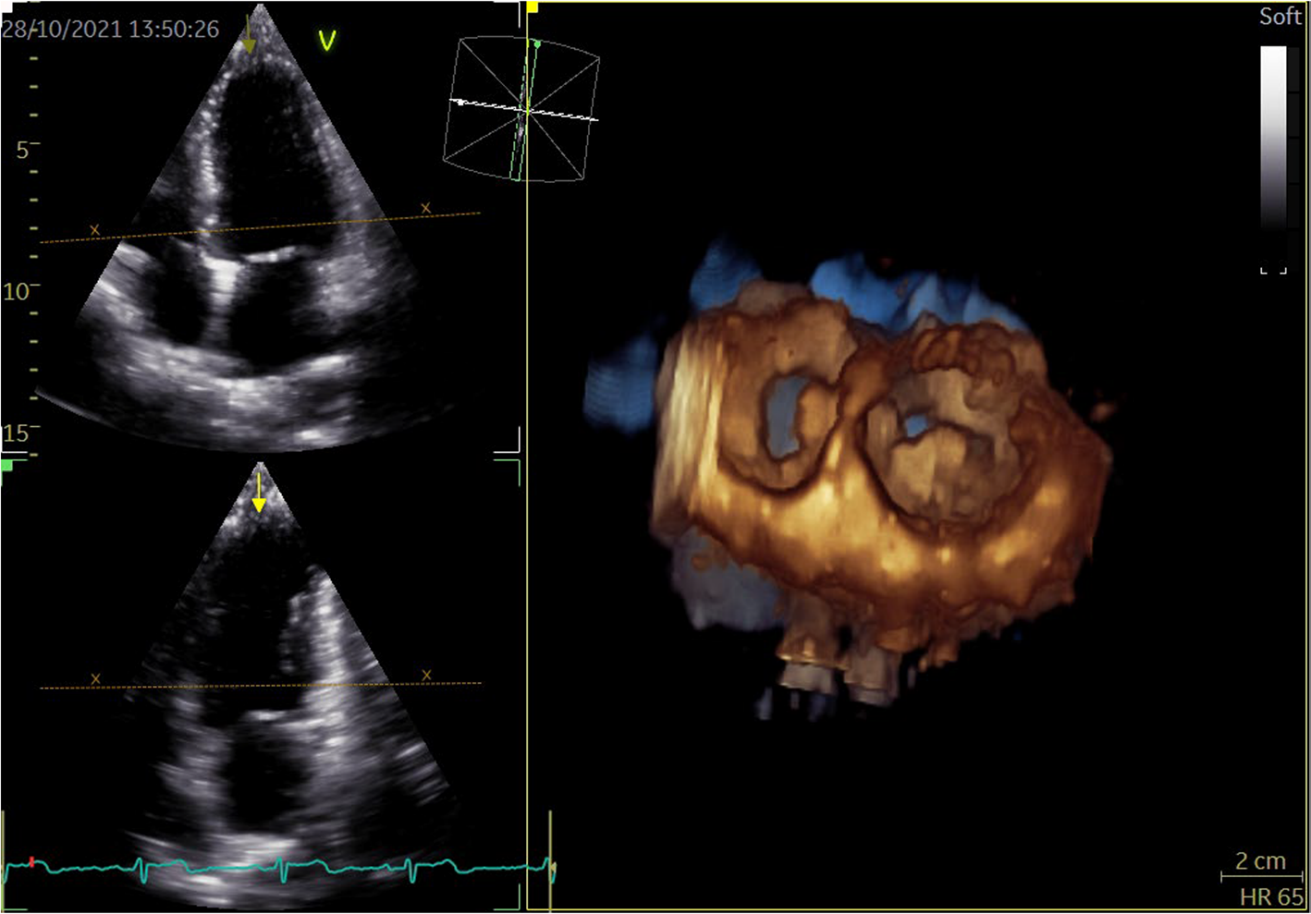
LA and LV full-volume images were obtained in apical four-chamber views, and high-quality 3D images of 4–6 cardiac cycles were recorded during sinus rhythm. LA, left atrial; LV, left ventricular; 3D, three-dimensional

**Fig. 3:**
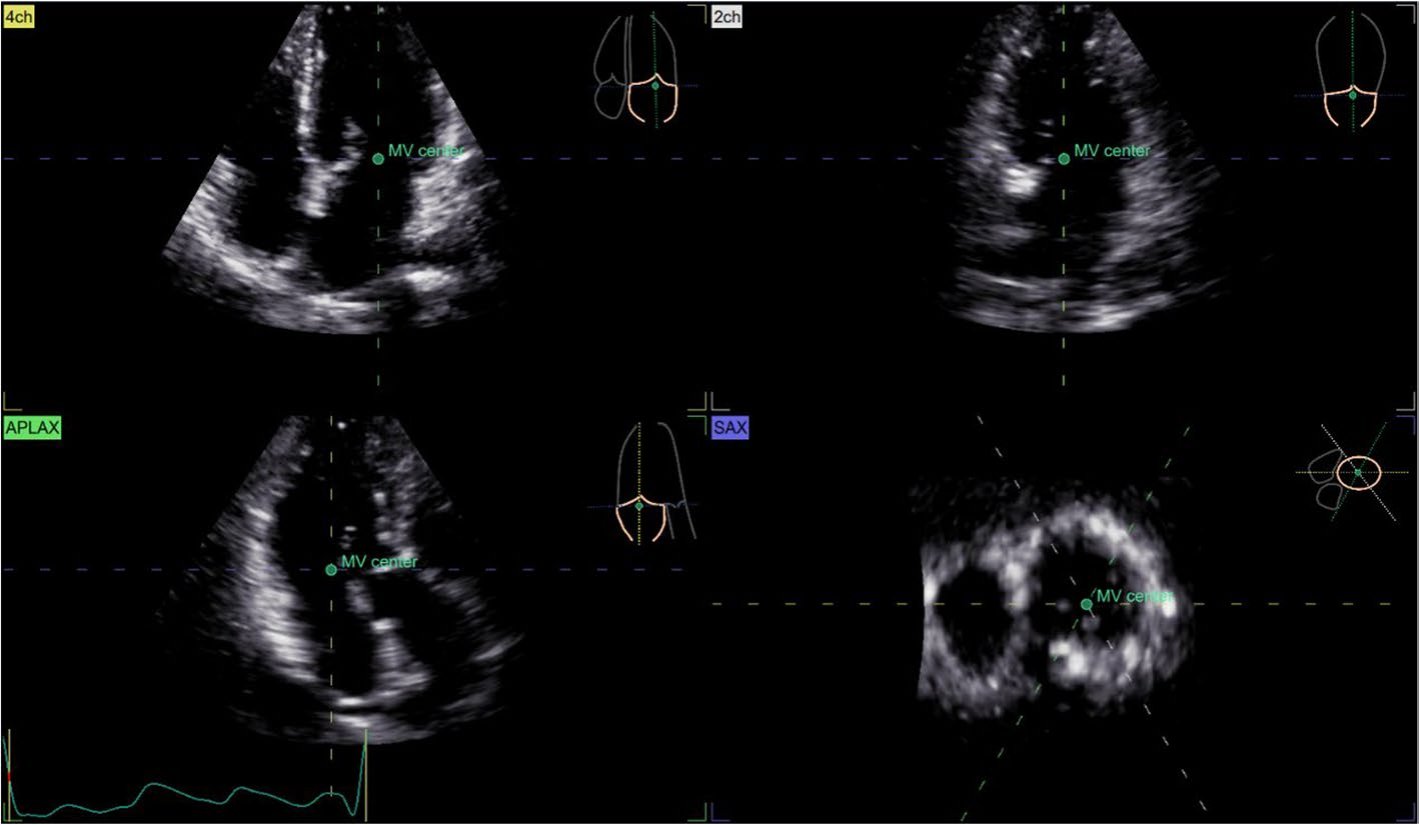
A landmark is precisely placed at the midpoint of the mitral annulus in three long-axis sections and one short-axis section

**Fig. 4:**
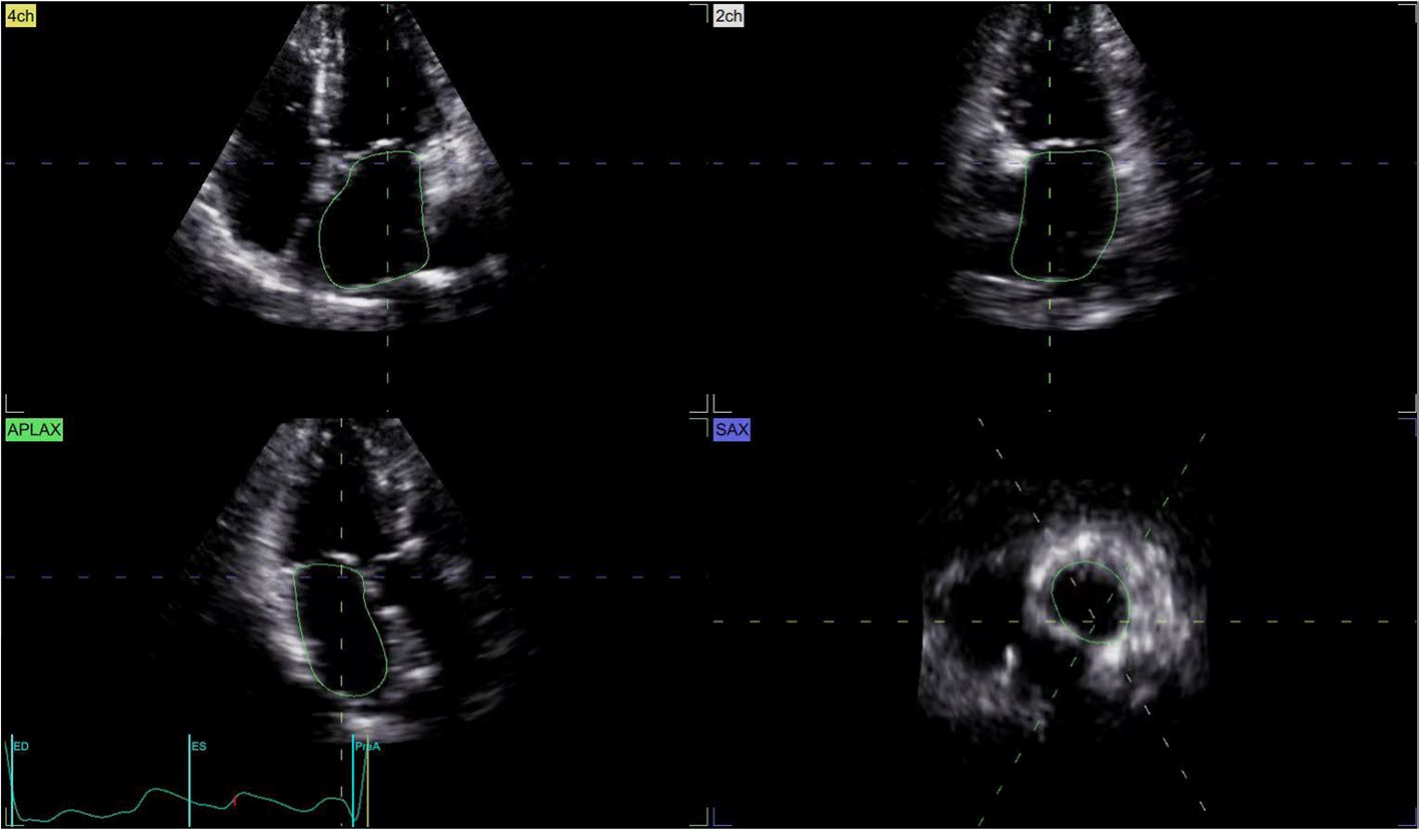
The system automatically tracks the left atrium's endocardial border and its motion throughout the cardiac cycle

**Fig. 5:**
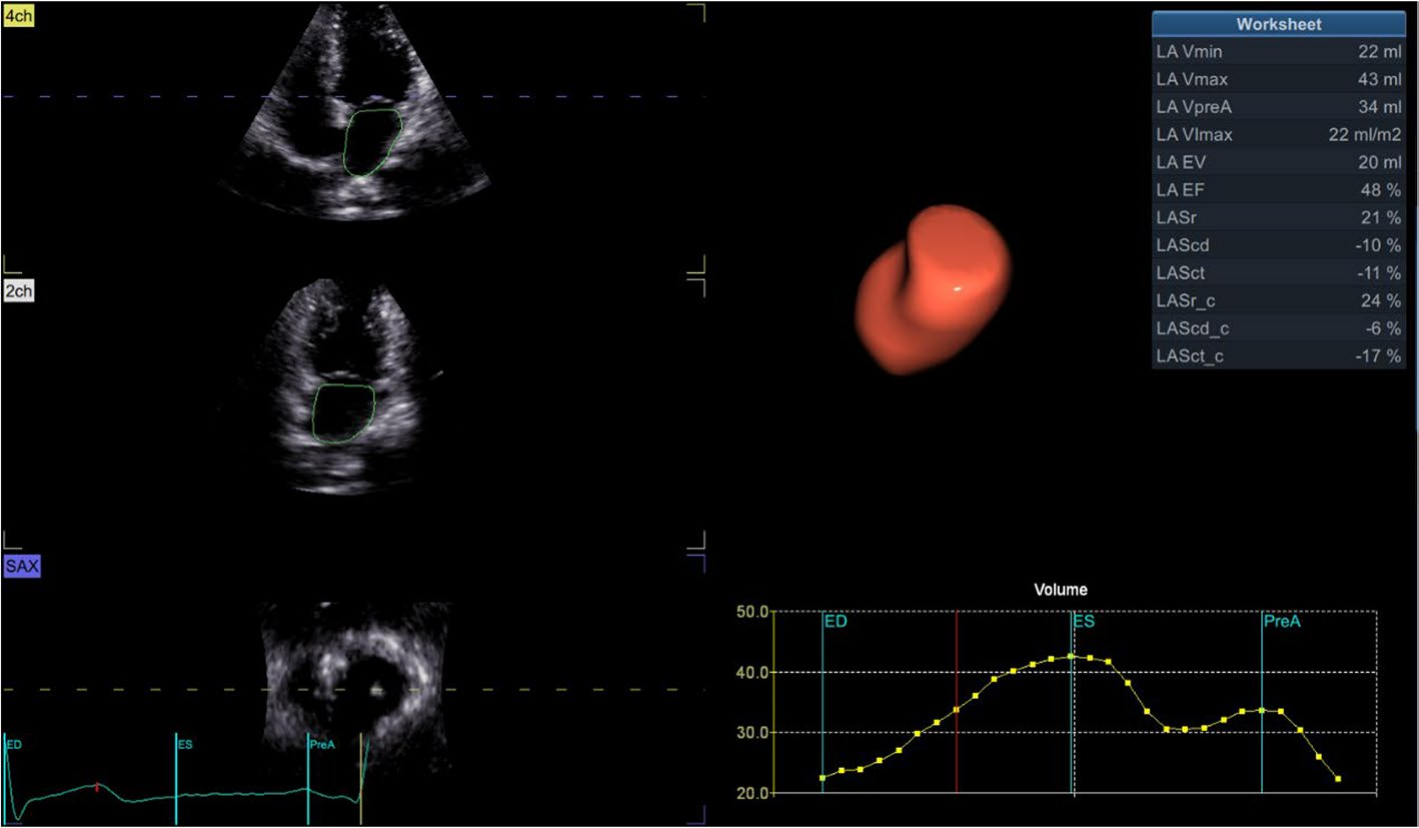
3D LA parameters were generated automatically by Echopac software. 3D, three-dimensional;LA, left atrial

LA and LV full-volume images were obtained in apical four-chamber views at a frame of 42/s. High-quality 3D images of 4–6 cardiac cycles were recorded during SR (Fig. 2). All images were transferred into EchoPAC software. “4D Auto LVQ” in “Cardiac” mode was selected, and the midpoint position of the LV apex and mitral annulus were identified manually at the end of diastole and systole. The software automatically delineated the boundary between the endocardium and epicardium of LV, apical four-chamber, three-chamber, two-chamber views, and three short-axis views were obtained automatically. Software then generated a myocardial 17-segment model and calculated the 3D parameters: left ventricular global longitudinal strain (LVGLS), left ventricular global circumferential strain (LVGCS), left ventricular global area strain (LVGAS), left ventricular global radial strain (LVGRS). A landmark is positioned at the midpoint of the mitral annulus, following a process similar to that for the left ventricle. Subsequently, the system automatically tracks the left atrium’s endocardial border and its motion throughout the cardiac cycle, allowing for manual adjustments when necessary (Figs. 3 and 4). The software automatically analyze and obtain the measured LA volume, LAVpreA (left atrial pre systolic volume), LAEF(left atrial ejection fraction), 3D LA parameters including reservoir, conduit as well as contract phase, LASr (left atrial reservoir strain), LAScd (left atrial conduit strain), LASct (left atrial contraction strain), LASr-c (left atrial reservoir circumferential strain), LAScd-c(left atrial conduit circumferential strain), LASct-c(left atrial contraction circumferential strain) (Fig. 5).

### Reproducibility

The intra- and inter-observer reliabilities of LASr were evaluated in a cohort of 20 randomly selected subjects using Bland-Altman plots. The same operator conducted measurements and repeated the process to assess intra-observer variability after a two-week interval. For inter-observer variability assessment, another experienced operator independently replicated the analysis in the same group of patients.

### Statistical analysis

The statistical analyses were carried out with SPSS 23.0 software (IBM Corp, Armonk, NY). For normally distributed continuous variables, an independent-sample t-test was used for comparison within the inter-group, non-normally distributed continuous variables between the two groups were compared by the Mann-Whitney U test. Categorical variables were performed using the χ2 test. Continuous variables were shown to be mean ± standard deviation or *P*50 (*P*25 and *P*75). Categorical data were denoted as percentages. Demographic data, along with two-dimensional and 3DSTE parameters, were employed to predict the probability of recurrence in patients with AF during the follow-up period using univariate regression analysis. Further multivariate regression analysis was conducted on variables for which the significance level of the univariate logistic regression was ≤ 0.2. The area under the curve(AUC) and cut-off values for parameters that exhibited independent predictive value were determined by receiver operating characteristic (ROC) curve. *p*-values < 0.05 showed statistical significance.

## Results

Baseline demographic data are characterized in Table 1. 109 PAF patients were included with 31 patients recurrencce and 78 maintained SR after one year period follow-up. The medians with interquartile ranges was represented as[12.2(12.0,12.6) versus 12.2(12.0,12.8)]. Recurrence patients having cases of hypertension 45.2%, diabetes mellitus 19.4%, coronary artery disease 9.7%, and cerebral embolism 3.2%. Age, gender, BMI and heart rate exhibited no significant differences between the recurrence and patients staying SR. No statistically significant difference was exhibited in the drug treatments administered to patients with PAF, which encompassed anticoagulant therapy, antiarrhythmic drugs, and antihypertensive medications (all *p* > 0.05).

**Table 1:** Comparison of patient demographic and clinical data

Variables	SR(*N* = 78)	Recurrence(*N* = 31)
Age(years)	61.8 ± 9.2	63.6 ± 7.1
Male(%)	47(60.3)	18(58.1)
BMI(kg/m²)	22.4 ± 2.3	22.8 ± 2.4
HR(beat/min)	74.1 ± 6.8	72.6 ± 5.5
Follow-up time(months)	12.2(12.0,12.6)	12.2(12.0,12.8)
CHA2DS2-VASc Score	1(0,2)	2(0,2)
Hypertension(%)	39(48.7)	14(45.2)
Diabetes mellitus(%)	10(12.8)	6(19.4)
Coronary artery disease(%)	11(14.1)	3(9.7)
Cerebral embolism(%)	5(6.4)	1(3.2)
Smoking(%)	21(26.9)	10(32.3)
Alcohol(%)	25(32.1)	12(38.7)
Anticoagulant therapy(%)	42(53.9)	21(67.7)
AAD(%)	59(75.6)	23(74.2)
ACEI/ARBs(%)	28(35.9)	13(42.0)
Statin (%)	12(38.7)	6(19.4)
Beta-blockers(%)	38(48.7)	20(64.5)
SGLT2 inhibitors(%)	2(2.6)	0
Calcium channel blockers(%)	18(23.1)	11(35.5)

*SR* sinus rhythm, *BMI* body mass index, *HR* heart rate, *AAD* antiarrhythmic drug, *ACEI* angiotensin converting enzyme, *ARBs* angiotensin II receptor blockers, *SGLT2 inhibitors* sodium-glucose cotransporter 2 inhibitors, 1mmHg=0.133kPa

Table 2 displays a comprehensive array of 3DSTE parameters among patients categorized by recurrence status before and after RFCA. Notably, LVGLS and LVGAS demonstrated a statistically significant increase in patients who maintained SR after ablation (*p* < 0.05). Conversely, the remaining echocardiography parameters did not exhibit notable significance. On the contrary, conventional parameters and LV 3D strains displayed a lack of discernible differentiation in patients experiencing recurrence.

**Table 2:** Comparison of Echocardiographic Parameters in PAF patients maintaining SR versus those with recurrence

Variables	Maintain SR		Recurrence	
	Before-RFCA *N* = 78	After-RFCA *N* = 78	Before-RFCA *N* = 31	After-RFCA *N* = 31
LAD	36.3 ± 4.7	34.9 ± 4.2	36.8 ± 5.6	35.4 ± 4.3
LAVI	28.1 ± 5.9	27.2 ± 7.2	30.1 ± 5.2	31.8 ± 6.9
LVEDd	47.0 ± 3.6	47.3 ± 3.8	48.0 ± 3.5	48.3 ± 4.8
LVEDs	29.5 ± 2.5	29.0 ± 3.5	30.0 ± 3.2	29.4 ± 3.1
LVEDV	94.6(84.7, 107.3)	93.0(85.0, 104.6)	92.3 ± 11.1	93.4 ± 8.8
LVESV	37.2 ± 6.6	38.1 ± 5.7	37.1 ± 6.4	39.3 ± 4.5
LVSV	57.6 ± 8.9	55.9 ± 8.5	55.2 ± 8.6	54.0 ± 7.5
LVEF	60.8 ± 4.9	59.4 ± 4.1	59.7 ± 5.1	57.8 ± 4.3
E	79.2(68.7, 88.4)	77.3(67.8, 89.4)	80.1 ± 13.2	75.9 ± 9.0
e’	8.4(7.5, 9.7)	8.9(7.9, 10.4)	8.0(7.2, 9.2)	7.9(7.2, 8.9)
E/e’	9.3 ± 1.3	8.8 ± 1.7	9.4 ± 1.8	9.6 ± 1.7
LVGLS	−17.0(−21.0, −13.0)	−19.0(−22.0,−16.0)^∗^	−16.0 ± 4.5	−17.3 ± 5.0
LVGCS	−19.0(−22.0, −16.0)	−20.0(−22.0, −17.8)	−19.0(−20.0, −17.0)	−18.0−22.0, −17.0)
LVGAS	−29.1 ± 4.5	−31.1 ± 4.2^∗∗^	−27.9 ± 4.0	−28.9 ± 3.6
LVGRS	46.1 ± 8.4	47.9 ± 7.5	43.7 ± 9.5	45.5 ± 5.8

*PAF* paroxysmal atrial fibrillation, *RFCA* radiofrequency catheter ablation, *SR* sinus rhythm, *LAD* left atrial diameter, *LAVI* left atrial volume index, *LVEDd* left ventricular end-diastolic diameter, *LVEDs* left ventricular end-systolic diameter, *LVEDV* left ventricular end-diastolic volume, *LVESV* left ventricular end-systolic volume, *LVSV* left ventricular stroke volume, *LVEF* left ventricular ejection fraction, *E* peak early diastole mitral flow velocity, *e* mitral annular septal diastolic velocity, *LVGLS* left ventricular global longitudinal strain, *LVGCS* left ventricular global circumferential strain, *LVGAS* left ventricular global area strain; LVGRS, left ventricular global radial strain

^∗^*p* < 0.05; ^∗∗^*p* < 0.01; ^∗∗∗^*p* < 0.001

Table 3 compares 2D echocardiographic and 3D strain parameters of the left ventricle before and after RFCA for patients with sustained SR and those who relapsed during follow-up. There were no significant differences in echocardiography parameters before RFCA between the recurrence and SR maintenance groups. However, patients with recurrence exhibited higher LAVI and E/e’ compared to those without recurrence (*p* < 0.05) after RFCA. Additionally, e’ was significantly lower in the recurrence group than in the SR maintenance group. While LVEF showed no statistically significant difference between the two groups, LV systolic function declined in patients with recurrence after RFCA. LVGLS and LVGAS values were notably lower in the recurrence group compared to the no-recurrence group, with no pre-RFCA differences.

**Table 3:** Comparison of echocardiographic in patients with PAF before and 1 year after RFCA

variables	Before-RFCA		Post-RFCA	
	Maintain SR *N* = 78	Recurrence *N* = 31	Maintain SR *N* = 78	Recurrence *N* = 31
LAD	36.3 ± 4.7	36.8 ± 5.6	34.9 ± 4.2	35.4 ± 4.3
LAVI	28.1 ± 5.9	30.1 ± 5.2	27.2 ± 7.2	31.8 ± 6.9^∗∗^
LVEDd	47.0 ± 3.6	48.0 ± 3.5	47.3 ± 3.8	48.3 ± 4.8
LVEDs	29.5 ± 2.5	30.0 ± 3.2	29.0 ± 3.5	29.4 ± 3.1
LVEDV	94.6(84.7,107.3)	91.7(83.7,100.2)	93.9 ± 11.9	93.4 ± 8.8
LVESV	37.2 ± 6.6	37.1 ± 6.4	38.2 ± 5.7	39.3 ± 4.5
LVSV	57.6 ± 8.9	55.2 ± 8.6	55.9 ± 8.5	54.0 ± 7.5
LVEF	60.8 ± 4.9	59.8 ± 5.1	59.4 ± 4.1	57.8 ± 4.3
E	79.2(68.7, 88.4)	79.5(68.0, 89.6)	77.9 ± 13.1	75.9 ± 9.0
e’	8.4(7.5, 9.7)	8.0(7.2, 9.2)	9.0 ± 1.5	8.0 ± 1.2^∗∗^
E/e’	9.3 ± 1.3	9.4 ± 1.8	8.8 ± 1.7	9.6 ± 1.7^∗^
LVGLS	−17.5 ± 5.2	−16.0 ± 4.5	−19.0(−22.0,−16.0)	−17.0(−20.0,−14.0) ^∗^
LVGCS	−19.0(−22.0,16.0)	−19.0(−20.0,−17.0)	−20.0(−22.0,−17.8)	−18.0(−22.0,−17.0)
LVGAS	−29.1 ± 4.5	−27.9 ± 4.0	−31.1 ± 4.2	−28.9 ± 3.6^∗^
LVGRS	46.1 ± 8.4	43.7 ± 9.5	47.9 ± 7.5	45.5 ± 5.8

*PAF* paroxysmal atrial fibrillation, *RFCA* radiofrequency catheter ablation, *SR* sinus rhythm, *LAD* left atrial diameter, *LAVI* left atrial volume index, *LVEDd* left ventricular end-diastolic diameter, *LVEDs* left ventricular end-systolic diameter, *LVEDV* left ventricular end-diastolic volume, *LVESV* left ventricular end-systolic volume, *LVSV* left ventricular stroke volume, *LVEF* left ventricular ejection fraction, *E* peak early diastole mitral flow velocity, *e* mitral annular septal diastolic velocity, *LVGLS* left ventricular global longitudinal strain, *LVGCS* left ventricular global circumferential strain, *LVGAS* left ventricular global area strain; LVGRS, left ventricular global radial strain

^∗^*p* < 0.05; ^∗∗^*p* < 0.01

Furthermore, with the exception of LAVpre and LAEF, all 3D LA strains exhibited a statistical increase in patients with PAF in comparison to pre-RFCA and post-RFCA who did not experience recurrence. In contrast, LA volume and strain parameters showed no significant differences in recurrent patients when comparing pre-RFCA and post-RFCA assessments (Table 4).

**Table 4:** Comparison of 3DSTE LA Parameters in patients maintaining SR and Recurrence

Variables	Maintain SR		Recurrence	
	Before-RFCA *N* = 78	After- RFCA *N* = 78	Before-RFCA *N* = 31	After-RFCA *N* = 31
LAVpreA	38.3 ± 11.3	40.2 ± 7.6	36.5 ± 13.1	41.8 ± 8.0
LAEF	45.5(39.8, 52.0)	48.5(43.0, 54.0)	43.2 ± 8.6	43.9 ± 5.9
LASr	17.0(14.0, 21.0)	21.0(17.0, 24.0) ^∗∗∗^	14.2 ± 4.2	14.0 ± 3.8
LAScd	−9.0(−11.3, −7.0)	−11.0(14.0, −9.0) ^∗∗∗^	−8.6 ± 2.6	−8.9 ± 3.1
LASct	−8.0(−10.0, −5.0)	−9.0(−11.0, −6.0) ^∗^	−6.4 ± 2.4	−6.6 ± 2.6
LASr-c	16.0(12.0, 20.0)	20.0(16.0, 25.3) ^∗∗∗^	14.0 ± 3.6	14.7 ± 5.2
LAScd-c	−8.0(−10.0, −6.0)	−10.0(−12.0, −8.0) ^∗∗∗^	−7.0(−9.0,−5.0)	−6.0(−8.0,−4.0)
LASct-c	−10.0(−11.3, −7.8)	−11.0(−13.3, −9.0) ^∗∗^	−8.8 ± 2.6	−7.8 ± 2.6

*3DSTE* Three-dimensional speckle tracking echocardiography, *LA* left atrial, *SR* sinus rhythm, *LAVpreA* left atrial pre systolic volume, *LAEF* left atrial ejection fraction, *LASr* left atrial reservoir strain, *LAScd* left atrial conduit strain, *LASct* left atrial contraction strain, *LASr-c* left atrial reservoir circumferential strain, *LAScd-c* left atrial conduit circumferential strain, *LASct-c* left atrial contraction circumferential strain

^*^p<0.05, ^**^p<0.01,^***^p<0.001

Table 5 illustrates the noteworthy disparities in 3D LA strains observed between the recurrent and non-recurrent groups both before and after the ablation at follow-up time. Specifically, Lasr and LAsr-c exhibited statistical distinctions between patients with SR and the recurrent group before the procedure. In contrast, subsequent to RFCA, all LA 3D strain parameters in SR maintenance group displayed significant deviations when compared with the recurrence group.

**Table 5:** Comparison of 3DSTE LA Parameters before and 1 year after RFCA at Follow-up

Variables	Before-RFCA		Post-RFCA	
	Maintain SR *N* = 78	Recurrence *N* = 31	Maintain SR *N* = 78	Recurrence *N* = 31
LAVpreA	38.3 ± 11.3	36.5 ± 13.1	40.2 ± 7.6	41.8 ± 8.0
LAEF	46.0 ± 8.0	43.2 ± 8.6	48.5(43.0, 54.0)	44.0(40.0,49.0) ^∗∗^
LASr	17.4 ± 4.7	14.2 ± 4.3^∗∗^	21.0(17.0, 24.0)	15.0(10.0,16.0) ^∗∗∗^
LAScd	−9.0(−11.3,−7.0)	−8.0(−10.0,−7.0)	−11.0(−14.0, −9.0)	−8.0(−12.0,−7.0) ^∗∗^
LASct	−8.0(−10.0,−5.0)	−6.0(−9.0,−5.0)	−9.0(−11.0,−6.0)	−7.0(−9.0, −5.0) ^∗∗^
LASr-c	16.0(12.0, 20.0)	15.0(11.0,17.0) ^∗^	20.6 ± 6.5	14.7 ± 5.2^∗∗∗^
LAScd-c	−8.0(−10.0, −6.0)	−7.0(−9.0, −5.0)	−10.0(−12.0, −8.0)	−6.0(−8.0, −4.0) ^∗∗∗^
LASct-c	−9.6 ± 3.0	−8.8 ± 2.6	−11.0(−13.3, −9.0)	−8.0(−10.0,−6.0) ^∗∗∗^

*3DSTE* Three-dimensional speckle tracking echocardiography, *LA* left atrial, *RFCA* radiofrequency catheter ablation, *SR* sinus rhythm, *LAVpreA* left atrial pre systolic volume, *LAEF* left atrial ejection fraction, *LASr* left atrial reservoir strain, *LAScd* left atrial conduit strain; LASct, left atrial contraction strain, *LASr-c* left atrial reservoir circumferential strain, *LAScd-c* left atrial conduit circumferential strain; LASct-c, left atrial contraction circumferential strain

^*^p<0.05, ^**^p<0.01,^***^p<0.001

In our study, a total of 78 patients maintained SR during the postoperative follow-up period. Subsequently, multivariate regression analyses were conducted, focusing on indicators with *p*-values ≤ 0.2 from the univariate regression analyses. The results indicated that LASr was an independent predictor for the maintenance of stable SR one year after RFCA. [odds ratio (OR), 1.19, 95% confidence interval (CI), 1.05–1.35, *p* = 0.005] (Table 6).

**Table 6:** Results of Univariate and Multivariate Regression Analysis to predict recurrence of AF after RFCA

Variables	Univariate analysis		Multivariate analysis	
	OR(95%CI)	*p* value	OR(95%CI)	*p* value
LAVI	0.94(0.87–1.01)	0.10	0.92(0.84–1.01)	0.10
LVGLS	0.94(0.86–1.03)	0.16	0.96(0.87–1.06)	0.41
LVGAS	0.94(0.85–1.03)	0.19	0.97(0.87–1.08)	0.57
LAEF	1.05(0.99–1.10)	0.10	1.06(0.99–1.13)	0.09
LASr	1.17(1.06–1.30)	0.003	1.19(1.05–1.35)	0.005
LAScd	0.90(0.78–1.05)	0.19	0.82(0.67–1.01)	0.06
LASct	0.86(0.73–1.02)	0.08	0.82(0.66–1.02)	0.08
LASr-c	1.11(1.00–1.23)	0.04	1.05(0.94–1.19)	0.39
LAScd-c	0.86(0.73–1.01)	0.06	0.85(0.71–1.03)	0.10
LASct-c	0.90(0.78–1.05)	0.18	1.09(0.90–1.32)	0.36

*AF* atrial fibrillation, *RFCA* radiofrequency catheter ablation, *OR* odds ratio, *CI* confidence interval, *LAVI* left atrial volume index, *LVGLS* left ventricular global longitudinal strain, *LVGAS* left ventricular global area strain, *LAEF* left atrial ejection fraction, *LASr* left atrial reservoir strain, *LAScd* left atrial conduit strain, *LASct* left atrial contraction strain, *LASr-c* left atrial reservoir circumferential strain, *LAScd-c* left atrial conduit circumferential strain, *LASct-c* left atrial contraction circumferential strain

**Fig. 6:**
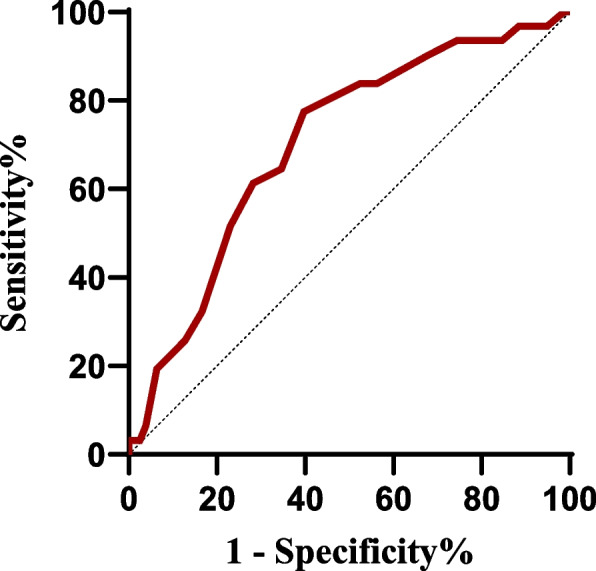
ROC curves for LASr to predict recurrence during the follow-up period after RFCA in PAF patients. LASr, left atrial reservoir strain; RFCA, radiofrequency catheter ablation; PAF, paroxysmal atrial fibrillation; ROC, receiver operating characteristic

The results of the receiver operating characteristic(ROC) curve indicated that the area under the curve(AUC) for LASr in assessing the risk of recurrence after RFCA in patients with PAF was 0.70 ( 95% CI 0.60 ~ 0.81, *P* = 0.001), with a sensitivity of 77.4% and a specificity of 60.9% (Fig. 6). The calculated cutoff value for LASr was 16.5%.

**Fig. 7:**
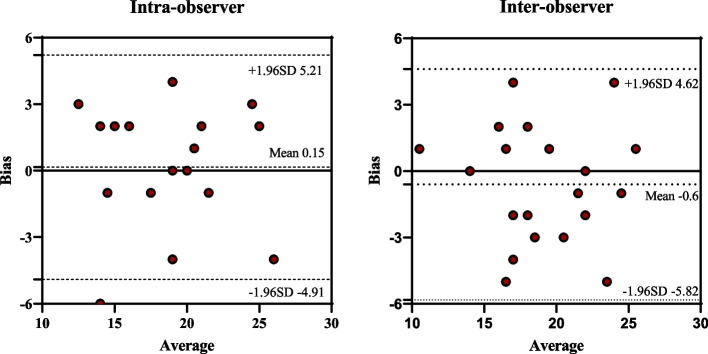
Bland-Altman plots for evaluation of LASr in the intra- and inter-observer reliabilities. LASr, left atrial reservoir strain

Bland-Altman plots was conducted to assess the intra- and inter-observer reliabilities of LASr (Fig. 7). The mean intra-observer bias for LASr was 0.18 (95% CI, −4.91–5.21), the mean inter-observer bias was − 0.6(95% CI, −5.82-4.62).

## Discussion

We evaluated the changes in LA myocardium mechanics in drug-refractory PAF patients before and one year follow-up time after RFCA. Our findings were:1. 3DSTE effectively assessed LA structure and function changes before and after RFCA. 2. Patients maintaining SR with PAF post-RFCA showed significant improvements in LA volumetric parameters and deformational capacity compared to those with recurrence. Moreover, patients who remained in SR demonstrated substantial recovery in LA reservoir, conduit, and booster pump function post-procedure, in contrast to their pre-procedural condition. 3. It is evident that pre-procedural assessment of LA strain is vital for identifying potential recurrences in patients with PAF after undergoing ablation.

While RFCA is frequently chosen as the primary treatment option, it remains associated with a noteworthy recurrence rate [12]. Hence, it is essential to preoperatively assess the likelihood of AF recurrence in patients undergoing RFCA. This assessment aids in selecting appropriate treatment modalities to enhance long-term prognostic outcomes. In this study, we observed that 28.4% of the 109 PAF patients experienced recurrence during the one-year follow-up period, which consistent with findings from a larger cohort study, where 71% of patients with PAF elimination was achieved [13]. Recent studies have highlighted mechanisms of recurrence, including the electrical reconnection of previously isolated pulmonary veins and the development of atrial fibrosis [14]. Atrial fibrosis plays a crucial role in AF progression, and its development is attributed to frequent mechanical of left atrial (LA) fibrosis stretch-induced alterations in electrical activity, which subsequently affect the electrical and mechanical properties of atrial myocytes. These changes lead to both electrical and structural remodeling of the left atrium [15].Current methods for assessing atrial fibrosis in AF include cardiac magnetic resonance imaging (CMRI), echocardiographic strain imaging, and the use of biomarkers. Research has demonstrated correlations among these three assessment methods [16]. In contrast to previous investigations, our study employed 3D-STE to construct a comprehensive 3D model of the heart. This approach facilitated precise quantification of myocardial deformation and enabled a comprehensive assessment of structural and functional alterations in the left atrium.

Our findings suggest a significant increase in LA deformation capacity among patients who maintained SR during a one-year follow-up, indicating improved LA reservoir, contractile, and conduit function. Moreover, studies have demonstrated an increase in the peak longitudinal LA strain rate during the reservoir and conduit phases, while other LA strain parameters remained unchanged at 12 months following successful ablation procedures [17].This synchronized atrial contraction, achieved after RFCA, not only leads to long-term restoration of cardiac output but also contributes to the reversal of atrial electrical and structural remodeling, resulting an augmentation of the integrated phases of the left atrium [18, 19].

Our echocardiographic assessment revealed that patients with PAF who restored SR after RFCA showed a statistically significant differences in LA size and function compared to those experiencing recurrence. A comparative study of LA function following RFCA in 90 patients with drug-refractory PAF revealed distinct patterns. Patients experiencing recurrence at one year postoperatively demonstrated significantly larger LA volume and reduced atrial function compared to those who maintained SR postoperatively, consistent with our own study’s findings. Notably, recurrent patients displayed a further decline in LA function relative to their preoperative state [20]. However, in our study, a statistically significant difference was not observed in LA function between recurrent patients’ preoperative and postoperative states. This divergence may be partly due to the limited sample size of recurrent cases and the potential dynamic changes in LA function post-RFCA. For a more comprehensive assessment, further categorization of recurrence, distinguishing between early and long-term recurrences based on the duration of AF, may be necessary to accurately evaluate left atrial function in these cases.

Several studies found that LA diameter, volume size, type of AF, demographic and serum markers are important factors for the recurrence after RFCA [7, 21]. Numerous studies have underscored the significance of LA storage function as a predictive factor for recurrence following radiofrequency ablation of AF [22]. The study conducted by Barbier et al. [23]identified two phases of left atrium storage function: an early phase associated with diastole following the previous left atrium contraction and a later phase linked to the contraction of longitudinally oriented LV myocardial fibers and LV stiffness. Their multiple regression analysis led to the conclusion that LA reservoir function stood as an independent predictor of cardiac output. Our study demostrated parameters assessing LA function via 3DSTE were not only sensitive to changes before and after RFCA but also served as predictors for recurrence during the follow-up period. Further multivariate regression analysis identified LASr as an independent predictor for PAF recurrence following RFCA. According to the ROC curve analysis, LASr < 16.5% indicates recurrence following RFCA during the follow-up period.

3D speckle tracking analysis of LA strain also encompasses the assessment of the contractile capacity of myocardial fibers in the circumferential direction. LASct has been proposed as a significant predictor of recurrence following AF ablation [24]. However, previous studies primarily focused on patients with long-standing persistent AF who could not maintain SR during image acquisition. These patients exhibited markedly diminished or absent LA systolic function, leading to notable differences in LASct values compared to those who did not experience recurrences. A statistically significant difference was observed when comparing different groups, while our study found that circumferential strain did not independently predict AF recurrence. Further research on this parameter is expected for a more comprehensive understanding in the future.

The left atrium and left ventricle engage in intricate interactions, exerting mutual influence at distinct phases of the cardiac cycle. Augmented LA storage capacity serves to sustain interatrial pressure, augment left ventricular blood volume, and enhance compliance. Extant literature substantiates the presence of a correlation between LA strain and left ventricular strain [25–27]. In our study, patients who maintained SR post-RFCA showed a significant increase in LVGLS and LVGAS, indicating recovery of left ventricular function while preserving LVEF. This improvement was observed when compared to preoperative and recurrent patients. Notably, our findings align with those of R. Ollivier et al. [28], who demonstrated significant enhancement in the assessment of longitudinal LV function using 2D-STE one year after RFCA for lone paroxysmal AF. We introduced LVGAS as a novel parameter that combines longitudinal and circumferential directions for LV function assessment. This novel approach proved to be more accurate and effective in evaluating 3D myocardial deformation.

These findings revealed that 3DSTE has great value and significance in evaluating subtle changes of LA myocardium in the subclinical phase, judging the development or prognosis of a disease, and prospecting broad clinical application value.

Our study presents several novel contributions to the field of AF management. By using 3DSTE, we were able to provide a more detailed evaluation of LA function than traditional two-dimensional methods. Additionally, we focused on patients with preserved LVEF—a subgroup not extensively studied before—allowing us to explore specific associations between LA strain parameters and AF recurrence. Additionally, we identified an independent 3DSTE predictor of AF recurrence from a comprehensive analysis that included clinical, biomarker, and echocardiographic parameters. These findings contribute novel insights that warrant further exploration in the field of atrial fibrillation management.

## Limitations

Our study’s limitations include a relatively small sample size, which may affect the generalizability of the results. A larger cohort, ideally involving multicenter trials, could provide a more comprehensive understanding and validation across diverse populations. The one-year follow-up may not sufficiently capture late recurrences of AF, which are crucial for assessing the long-term effectiveness of RFCA and cardiac remodeling. While early recurrences (within three months) are often due to transient inflammation from the ablation procedure, late recurrences are more indicative of the long-term efficacy of RFCA and structural remodeling in the heart. Extending the follow-up would help differentiate early transient recurrences from late ones that signal sustained efficacy. Additionally, the reliance on high-quality 3DSTE imaging presents challenges, especially in patients with suboptimal echocardiographic windows, and the use of different analysis software can introduce inconsistencies in strain measurements. AADs taken during the follow-up, the impact of different ablation techniques and lesion sets—like radiofrequency versus cryoablation—on long-term outcomes, including LA remodeling and recurrence may also impact recurrence rates potentially. Future studies should consider longer follow-up durations, standardized imaging software, AAD-free analyses, and explore how ablation modality and extent affect outcomes.

## Conclusions

RFCA demonstrates significant efficacy in the improvement of LA volume and function among patients diagnosed with drug-refractory PAF. LASr emerges as an independent predictor for post-ablation recurrence. 3DSTE has great value and significance in evaluating subtle changes of LA myocardium, judging the development or prognosis of PAF, and prospecting broad clinical application value.

## Data Availability

The datasets generated during the current study are not publicly available due to the restrictions by the Fourth Affiliated Hospital of Harbin Medical University but are available from the corresponding author on reasonable request.

## Abbreviations

2D: two dimensional
2DSTE: two-dimensional speckle tracking echocardiography
3D: three dimentional
3DSTE: Three-dimensional speckle tracking echocardiography
AAD: antiarrhythmic drug
ACEI: angiotensin converting enzyme
ACT: activated clotting time
AF: atrial fibrillation
ARBs: Angiotensin II Receptor Blockers
ATP: adenosine triphosphate
AUC: area under the curve
BMI: body mass index
CI: confidence interval
CMRI: cardiac magnetic resonance imaging
CPVI: circumferential pulmonary vein isolation
E: Peak early diastole mitral flow velocity
e ': mitral annular septal diastolic velocity
LVEDV: left ventricular end-diastolic volume
LVESV: left ventricular end-systolic volume
HF: heart failure
LA: left atrial
LAD: left atrial diameter
LAEF: left atrial ejection fraction
LAVpreA: left atrial pre systolic volume
LAScd: left atrial conduit strain
LAScd-c: left atrial conduit circumferential strain
LASct: left atrial contraction strain
LASct-c: left atrial contraction circumferential strain
LASr: left atrial reservoir strain
LASr-c: left atrial reservoir circumferential strain
LAVI: left atrial volume index
LV: left ventricular
LVEDd: left ventricular end-diastolic diameter
LVEDs: left ventricular end-systolic diameter
LVEF: left ventricular ejection fraction
LVGAS: left ventricular global area strain
LVGCS: left ventricular global circumferential strain
LVGLS: left ventricular global longitudinal strain
LVGRS: left ventricular global radial strain
OR: odds ratio
PAF: paroxysmal atrial fibrillation
RFCA: Radiofrequency catheter ablation
ROC: Receiver operating characteristic
SGLT2 inhibitors: sodium-glucose cotransporter 2 inhibitors
SR: SR
SV: Stroke volume
TTE: transthoracic echocardiography
